# Effectiveness of COVID-19 mRNA vaccine in preventing infection against Omicron strain: Findings from the Hiroshima Prefecture COVID-19 version J-SPEED for PCR center

**DOI:** 10.1371/journal.pgph.0003071

**Published:** 2024-04-17

**Authors:** Yui Yumiya, Kenya Kawanishi, Odgerel Chimed-Ochir, Eisaku Kishita, Aya Sugiyama, Junko Tanaka, Tatsuhiko Kubo

**Affiliations:** 1 Department of Public Health and Health Policy, Graduate School of Biomedical and Health Sciences, Hiroshima University, Hiroshima, Japan; 2 Medical Economics Division, Health Insurance Bureau, Ministry of Health, Labour and Welfare, Tokyo, Japan; 3 Department of Epidemiology, Infectious Disease Control and Prevention, Graduate School of Biomedical & Health Sciences, Hiroshima University, Hiroshima, Japan; 4 Medical Policy Office, Hiroshima University, Hiroshima, Japan; Yale School of Medicine: Yale University School of Medicine, UNITED STATES

## Abstract

**Background and purpose:**

Despite the widespread adoption of various preventive measures since the spread of COVID-19, there remains a lack of consensus on universally acknowledged best practices. However, the significance of vaccination has risen to prominence as a paramount preventive strategy. Numerous investigations have demonstrated vaccine effectiveness against the omicron strain in severe disease and symptomatic disease, however, the scope of research pertaining to vaccine effectiveness in preventing infection is presently limited. Therefore, the current study aimed to evaluate COVID-19 mRNA (Pfizer-BioNTech BNT162b2 or Moderna mRNA-1273) vaccine effectiveness in preventing infection.

**Methods:**

We conducted a test-negative case-control study using a dataset of 117,335 individuals. These data were collected through the COVID-19 J-SPEED form in the PCR center at Hiroshima Prefecture, Japan from 1 February to 17 March 2022. We estimated propensity score matching for vaccine status based on participants’ demographic characteristics. Subsequently, odds ratio was calculated from logistic regression to determine the association between vaccination status and test positivity rate adjusting for symptoms, exposure to close contact, and previous infection history. Vaccine effectiveness was defined as (1 –aORs) ×100%.

**Results:**

The PCR test positivity rates were 7.9%, 4.5%, and 2.8% for the non-vaccinated (non-vaccinated, vaccinated with a single dose, and vaccinated with two doses less than 14 days ago), vaccinated with two doses (vaccinated over 14 days ago), and three doses, respectively. In unadjusted and adjusted analyses, vaccine effectiveness of two doses against infection were 38.5% (95% confidence interval [CI]: 32.8%–43.8%) and 34.7% (95%CI: 28.4%–40.4%), respectively, compared to non-vaccinated group. Vaccine effectiveness of three doses were 33.8% (95%CI: 25.0%–41.5%) and 26.4% (95%CI: 16.4%–35.2%), respectively, compared to those vaccinated with two doses.

**Conclusions:**

These results illustrate the protective effect of mRNA vaccines against Omicron strain infections and emphasize the significance of completing the suggested vaccination schedule.

## Introduction

An outbreak of novel coronavirus infection began in Wuhan, China, in December 2019, subsequently spreading globally. Since then, a series of mutant strains have emerged. The Omicron BA.1 strain, which first emerged in South Africa at the end of November 2021, [1], swiftly disseminated worldwide, displacing previous strains such as Delta [1, 2]. To counter the emergence of a succession of mutant strains, countries have been promoting infection control measures, but there has been no convergence toward sharing best practices. However, vaccination stands as one of the most crucial preventive measures.

In Japan, the administration of third vaccine dose initiated in December 2021, coinciding with the replacement of Omicron strain by Delta [3], with priority given to healthcare workers and the elderly people. Subsequently, a fourth dose was began in May 2022 for individuals aged 60 or older, those aged 18 to 60 with underlying medical conditions, and those at a recognized high risk of serious illness upon coronavirus infection [4]. In response to the spread of infection with Omicron strain, the Japanese government expanded vaccine eligibility to healthcare workers and staff in elderly care facilities from July 2022. In Japan, most of vaccine types were administered: Pfizer Biontec BNT162b2 and Moderna mRNA-1273 [5].

Regarding vaccine effectiveness (VE), countries with more advanced vaccination programs than Japan have reported the VE against Omicron strain in preventing infection [6, 7], severity [8–10], symptomatic diseases [9, 11, 12], hospitalization [8, 13] and mortality [10]. Preventing infections, alongside averting severity, hospitalization, and mortality, becomes crucial for alleviating the burden on healthcare facilities and resources, and play crucial role in protecting the health and safety of their communities [14].

At our knowledge, few studies were conducted to study the VE, particularly effectiveness of booster vaccines, against Omicron strains in preventing COVID-19 infections in the general population [15–17]. However, repetitive evaluations of VE offer merits in identifying any gaps or challenges in VE across different populations or specific geographic areas [18]. The study’s context and findings contribute to the global understanding of VE. In addition, such repeated analyses contribute to build public trust in vaccination efforts. This fosters greater vaccine acceptance, bolstering infectious disease control [19]. International health organizations might need to adjust their guidance based on emerging data to ensure that vaccination strategies remain effective across different regions. Therefore, in our study, we aim to elucidate the VE against the Omicron strain. Our analysis relies on data collected at PCR centers in Hiroshima Prefecture, Japan from 1 February and 17 March 2022, a period dominated by the Omicron variant in Hiroshima Prefecture [20].

## Materials and methods

### Ethics statement

Approval for ethical review was obtained from Hiroshima University on 29 June 2021 (approval number: E-2508). This study was funded by the AMED (grant No.: JP20fk0108453h0001, JP21fk0108550h0001). The data used for the analysis is anonymized, ensuring the confidentiality of participants’ identities and personal information. The individuals who underwent PCR testing voluntarily participated in the study by answering to the questionnaire.

### Study design and data collection

This study design is a test-negative case-control study.

Hiroshima Prefecture, Japan introduced a standard data collection and reporting tool called Hiroshima Prefecture COVID-19 version J-SPEED form (hereinafter COVID-19 J-SPEED form) [20]. Original J-SPEED tool was developed following the lessons learned from the Great East Japan Earthquake in 2011 with the concept of simplifying and standardizing health data collection for near real-time situation [21, 22]. Modified for the COVID-19 context, this tool was employed across various settings, including public health centers [23], recuperation hotels, online treatment centers, oxygen centers, PCR centers [23], and hospitals.

In the current study, the data collected via COVID-19 J-SPEED form at PCR centers in Hiroshima Prefecture were used. The PCR center was established as a free access point for any asymptomatic Hiroshima Prefectural residents or travelers who had concerns about infection. Upon visiting the PCR center, they were requested to complete the COVID-19 J-SPEED form as a prerequisite for undergoing the PCR test. PCR test results are notified via phone for positive cases and SMS for negative results [24]. Although genome sequencing wasn’t performed within this study, Hiroshima Prefecture conducted genome analysis on COVID-19 patients during the study period, revealing that over 75% of cases were attributed to the “BA.1” strain and the remainder to “BA.2” [25]. Therefore, PCR-positive individuals in this study were assumed to be infected with the Omicron strain.

The COVID-19 J-SPEED form for PCR center includes 58 items, such as demographic information, occupation, symptoms, pre-existing diseases, vaccination history, previous infection history, exposure to close contact, adherence to COVID-19 preventive measures, and PCR test result. For the exposure to close-contact, we identified close contact through exposure to the “three Cs”—crowded places, close-contact settings, confined and enclosed spaces within 14 days or contact with a person who was tested PCR positive within 14 days. Since 1 April 2021, over one million people underwent PCR testing at PCR centers in Hiroshima Prefecture (As of July 5, 2022). Over time, the form was modified several times, and from 20 December 2021, a question on vaccination history was included in the questionnaire. Consequently, we used data from 1 February to 17 March 2022. Our study encompassed 117,335 individuals who underwent PCR testing at 18 PCR centers in Hiroshima Prefecture within the study period.

### Data analysis

We extracted information on demographics, vaccination history, previous infection history, PCR test results, and potential COVID-19 infection risk factors, including being a healthcare worker, the presence of pre-existing diseases (diabetes, hypertension, hyperlipidemia, rheumatism, cancer, angina pectoris, arrhythmia, heart disease, stroke, or any of the others), the presence of symptoms, and likelihood of close contact with infection source. Questions on vaccination status offered following options: non-vaccinated, vaccinated with a single dose, vaccinated with two doses less than 14 days ago, vaccinated with two doses over 14 days ago, and vaccinated with three doses. For analysis, we grouped the first three options together as ‘non-vaccinated’. This grouping based on the previous study reporting no significant difference in the effectiveness of mRNA vaccines (Moderna mRNA-1273) in preventing infection against Omicron strains after a single vaccination [25].

For the analysis, firstly, we descriptively analyzed the number of participants tested and those who got tested positive for each vaccination status category. Secondly, in order to ensure a balanced distribution of measured baseline covariates among individuals who were non-vaccinated, received two doses, and received three doses, we estimated propensity scores for vaccination status based on participants’ demographic characteristics including age, sex, occupation and pre-existing diseases with a logistic regression model. For matching, we utilized a 1:1 nearest-neighbor matching technique without replacement. The estimated propensity scores were used to match i) individuals who received two doses of vaccination with those who didn’t receive any vaccine (Model 1). This comparison is crucial for understanding the effectiveness of partial vaccination and informing public health measures, such as the need for additional doses or booster shots [26]; and ii) individuals who received three doses of vaccination with those who received two doses (Model 2). This comparison is also significant because due to the emergence of new variants and waning immunity over time, studying the VE of booster doses is essential and it helps assess the effectiveness of additional doses in enhancing immunity and providing protection against new variants [27]

C-statistics is calculated to assess the ability to correctly differentiate between the two groups (Model 1: 0.771; Model 2: 0.783). A caliper width of 0.2 standard deviations of the logit of the propensity score was used for matching [28]. Subsequently, a logistic regression was used to determine the association between vaccination status and test positivity rate. Adjusted odds ratios (aOR) and 95% confidence intervals (95% CI) were calculated. VE was defined as (1 –aORs) ×100%. SPSS statistical software (version 28; IBM, Armonk, NY, USA) was used for data analysis.

## Results

Table 1 shows the test positivity rates categorized by vaccination status and further grouped by age, sex and additional factors including occupation, pre-existing disease, symptoms, and exposure to close contacts. During the study period, a total of 117,335 individuals underwent PCR testing, yielding an average test positivity rate of 5.0 percent. The test positivity rate was higher among individuals who were not vaccinated (7.9%), in comparison to those who received two doses (4.5%) and three doses (2.8%). The group of individuals who had received three doses exhibited considerably lower test positivity rates across all age groups, both males and females, as well as within occupation groups—excluding medical professionals. Among medical professionals, those who had received two vaccine doses demonstrated a lower test positivity rate (3.0% with 54 cases) than those who had received three doses (3.3% with 130 cases).

**Table 1:** Summary of vaccination status and PCR test positivity rate by demographic variables and some potential risk factors.

	Non-vaccinated^1^			Vaccinated with 2 doses			Vaccinated with 3 doses			Total		
	N	No. of PCR+	PCR+ rate	N	No. of PCR+	PCR+ rate	N	No. of PCR+	PCR+ rate	N	No. of PCR+	PCR+ rate
**Total**	**26,205**	**2,065**	**7.9%**	**73,287**	**3,323**	**4.5%**	**17,843**	**503**	**2.8%**	**117,335**	**5,891**	**5.0%**
**Age group**												
0–9	9,230	753	8.2%	71	-	0.0%	7	-	0.0%	9,308	753	8.1%
10–19	4,813	377	7.8%	6,776	409	6.0%	151	5	3.3%	11,740	791	6.7%
20–39	6,410	541	8.4%	26,444	1,399	5.3%	4,069	161	4.0%	36,923	2,101	5.7%
40–59	4,418	311	7.0%	29,927	1,117	3.7%	6,586	167	2.5%	40,931	1,595	3.9%
60–79	1,206	73	6.1%	9,127	335	3.7%	6,225	146	2.3%	16,558	554	3.3%
80≤	128	10	7.8%	942	63	6.7%	805	24	3.0%	1,875	97	5.2%
**Sex**												
Male	13,893	1,123	8.1%	40,979	1,881	4.6%	7,118	194	2.7%	61,990	3,198	5.2%
Female Non_pregnant	12,051	915	7.6%	31,642	1,412	4.5%	10,576	308	2.9%	54,269	2,635	4.9%
Female Pregnant	228	19	8.3%	609	29	4.8%	136	1	0.7%	973	49	5.0%
**Risks**												
Non-Medical	25,703	2,035	7.9%	71,516	3,269	4.6%	13,939	373	2.7%	111,158	5,677	5.1%
Medical Professional	502	30	6.0%	1,771	54	3.0%	3,904	130	3.3%	6,177	214	3.5%
Absence of symptoms	23,624	1,424	6.0%	68,142	2,165	3.2%	16,981	361	2.1%	108,747	3,950	3.6%
Presence of Symptoms	2,581	641	24.8%	5,145	1,158	22.5%	862	142	16.5%	8,588	1,941	22.6%
Absence of pre-existing disease^2^	22,341	1,806	8.1%	55,904	2,651	4.7%	10,705	327	3.1%	88,950	4,784	5.4%
Presence of pre-existing disease	3,864	259	6.7%	17,383	672	3.9%	7,138	176	2.5%	28,385	1,107	3.9%
**Close contact** ^3^												
Absence	23,389	1,872	8.0%	63,859	3,038	4.8%	13,109	370	2.8%	100,357	5,280	5.3%
Presence	2,816	193	6.9%	9,428	285	3.0%	4,734	133	2.8%	16,978	611	3.6%
**Previous infection history**												
Absence	115,286	5,800	5.0%	25,854	2044	7.9%	71,719	3,256	4.5%	17,713	500	2.8%
Presence	2,049	91	4.4%	351	21	6.0%	1,568	67	4.3%	130	3	2.3%

N: Number of individuals visited PCR center

No. of PCR+: Number of individuals who got positive PCR test result

PCR+ rate: PCR test positivity rate

^1^Includes non-vaccinated, vaccinated with a single dose, and two doses with less than 14 days ago

^2^Includes diabetes, hypertension, hyperlipidemia, rheumatism, cancer, angina pectoris, arrhythmia, heart disease, stroke, or any of the others

^3^Visiting places that fall under the 3Cs within 14 days or Contact with a person who tested PCR positive within 14 days

**Fig 1:**
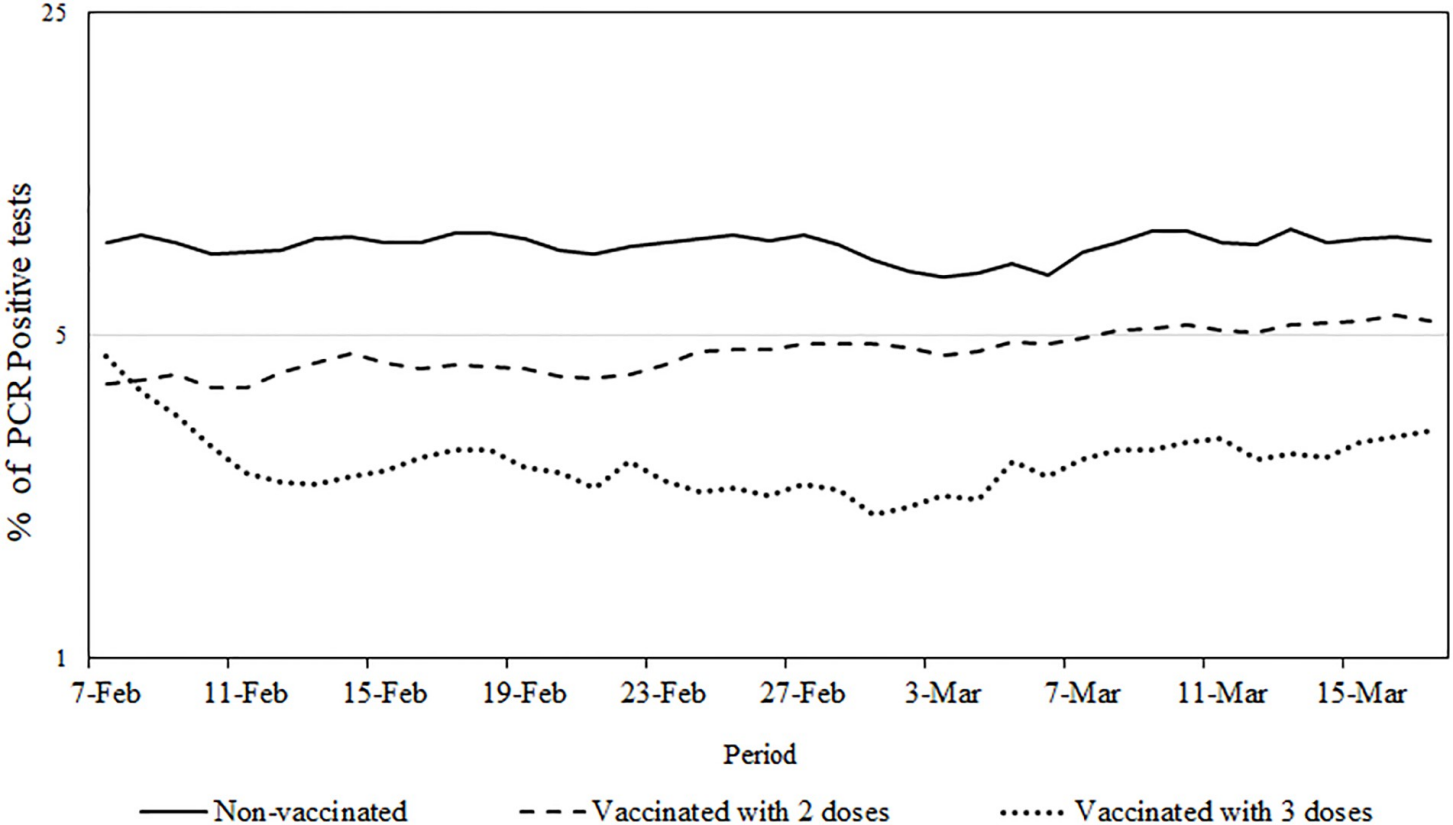
PCR test positivity rates by vaccination status. The x axis represents the study period; y axis represents the test positivity rate. Figure displays PCR positive rates for three distinct groups: non-vaccinated, vaccinated with two doses and vaccinated with third doses (booster).

Fig 1 illustrates a 7-day moving average of the test positivity rate among individuals categorized by their vaccination status: non-vaccinated, vaccinated with two doses, and vaccinated with three doses. Throughout the entire study duration, the lowest test positivity rate was consistently observed among individuals who received three doses of the vaccine. In contrast, the non-vaccinated group exhibited a comparatively higher test positivity rate.

Table 2 depicts the calculated vaccine effectiveness. In adjusted models, VE was 34.7% (95% confidence interval [CI]: 28.4%–40.4%) for individuals who received two doses of vaccine compared to the non-vaccinated group. Similarly, VE was 26.4% (95%CI: 16.4%–35.2%) for individuals who were vaccinated with three doses compared to those who were vaccinated with two doses.

**Table 2:** mRNA vaccine effectiveness administered in Hiroshima Prefecture, Japan.

Vaccination status	VE	95% Confidence Interval Interval	*P* value
**Model 1** *			
Non-vaccinated**	Reference		
2 doses (unadjusted)	38.5%	32.8–43.8%	< .001
2 doses***	34.7%	28.4–40.4%	< .001
**Model 2** *			
2 doses	Reference		
3 doses (unadjusted)	33.8%	25.0–41.5%	< .001
3 doses***	26.4%	16.4–35.2%	< .001

*To estimate the propensity score, we fitted a logistic regression model for vaccination status as a function of individual’s demographic factors including age, sex, occupation, and pre-existing diseases.

**Includes non-vaccinated, vaccinated with a single dose, and two doses with less than 14 days ago

***Adjusted for close contact (Visiting places that fall under the 3Cs within 14 days or Contact with a person who tested PCR positive within 14 days), symptoms PCR positive within 14 days), symptoms and previous infection history

## Discussion

In general, the study finding supports the effectiveness of mRNA vaccines in preventing infections caused by Omicron strains. The observed trends in VE reveal an effectiveness between individuals who received two doses in comparison to those with less than two doses. Similarly, the effectiveness was evident when comparing individuals who have had three doses against those with two doses.

While the study findings demonstrate a higher effectiveness of the third dose of the vaccine compared to the second dose, it’s crucial to interpret these results while taking certain situational factors into account. For example, the effectiveness of the second dose of the vaccine was already considered to have declined during the period of this study. The two-dose immunization coverage reached its peak (about 70%) in Hiroshima in November 2021 [29]; thus at least 4 months passed after the second vaccination. According to a study conducted in the U.S. on the efficacy of infection prevention against the Omicron strain in persons aged 18 years and older, two doses of the Moderna vaccine have been shown to be 44.0% effective in preventing infection after 14–90 days, with decreasing effect over time thereafter [15]. Similarly, another U.S. study that collected information on Pfizer vaccine recipients has reported a significant decline in the effectiveness of the second dose in preventing infection in persons aged 12 years and older, from 88% after one month to 47% after five to six months [30]. Furthermore, our result showed that the test positivity rate of elderly people over 80 years old who may have received the vaccine earlier than other generations, was highest among those who received two doses of vaccine. Thus, it is considered that during the study period, the infection prevention effectiveness of the second vaccination had already weakened and coincided with the time when the highly infectious Omicron strain began to spread. Therefore, it underscores the importance of monitoring the timing and intervals between vaccine doses. This highlights the need for regular assessment and potential adjustments to vaccination schedules.

In addition, the infection-preventive effect of the third dose vaccine was considered to be maintained during the period of this study. Previous study reported that the effectiveness of the third dose vaccination was 71.6% after 14–60 days and 47.4% after 61 days, indicating that the third vaccination temporarily restores the effectiveness of the vaccination in preventing infection [11]. Another nationwide representative study in Spain estimated 51.3% effectiveness of booster vaccines against infection up to 34 days after its administration [31]. UKHSA also estimated 45% (35% to 55%) effectiveness in preventing infection up to three months, but insisted there is currently little evidence, and the findings are not conclusive [11]. In this study, the third vaccination was administered within two months before the analysis, so that the infection-preventive effect was still maintained, potentially contributing to a reduced susceptibility to infection. However, a paradox emerged as healthcare workers who received the third vaccine dose exhibited a higher test-positive rate compared to healthcare workers who received the two doses. This disparity could be attributed to the heightened likelihood of breakthrough infections among medical professionals, owing to their increased exposure to concentrated contacts and patients, in contrast to other occupations. This observation suggest that certain professional groups may require tailored vaccination and preventive strategies. Such considerations could include additional protective measures for individuals with heightened exposure to the virus.

Our study’s notable strength lies in the comprehensive data collection conducted across all PCR centers in collaboration with the Hiroshima prefectural office. This extensive and unique epidemiological survey stands as a significant contribution, with no comparable large-scale study conducted in other prefectures of Japan. Moreover, the inclusion of detailed information encompassing occupation and various risk factors, such as the likelihood of close contact and pre-existing conditions, further enhances the robustness of our analysis. These factors were adjusted within the model to account for potential confounding influences.

There are certain limitations and challenges that warrant considerations. Firstly, our study did not permit the specific estimation of VE for individual vaccine types, booster types (heterologous or homologous), time-sensitive effectiveness (effectiveness at different intervals post-vaccination), or the interval between vaccine doses. This limitation stemmed from the absence of pertinent information in the questionnaire, including details regarding vaccine types and vaccination dates. Secondly, the generalizability of our results may be affected due to the nature of our study population. PCR test recipients who visited a PCR center may have a higher level of health consciousness compared to those who didn’t visit a PCR center. Additionally, it’s worth noting that a considerable proportion of the participants who underwent PCR testing at PCR centers were asymptomatic. This scenario potentially skews the representation of individuals with more pronounced symptoms, who may be more inclined to visit clinics rather than PCR centers. Therefore, the applicability of our findings should be interpreted with caution and consideration of these inherent limitations.

## Conclusion

These findings collectively underscore the protective impact of mRNA vaccination against Omicron strain infections and highlight the importance of completing the recommended vaccine dosing regimen. We believe that the repeated VE evaluations in this study will contribute to global understanding of VE and increasing public confidence in vaccination efforts. This would promote vaccine acceptance and strengthen new infectious disease control efforts in the future.
